# Supplementary material for: Improved annotation with de novo transcriptome assembly in four social amoeba species
Source: BMC Genomics. 2017 Jan 31;18:120. doi: 10.1186/s12864-017-3505-0 (PMC5282741; doi:10.1186/s12864-017-3505-0)
Supplement: Additional file 1: Figure S1. — TAGC plot for D. fasciculatum (A) and D. lacteum (B) before and after filtering. Each colour blob represents different taxa with unmatched transcripts are shaded in grey. The unannotated grey coloured transcripts after filtration set further filtered by high GC and low read coverage. This plot shows a major blob of transcripts that are annotated with the Dictyostelium fasciculatum species with high coverage and lower GC content. Other contaminations form E.coli, pseudomonas fluorescence and other species has also been highlighted with different colours. These contaminations clearly make different blobs with lower read coverage and high GC content. However, it’s good to see that there are some other transcripts that showing matched to dictyostelium discoideum- that clearly reflect the presence of some novel unannotated transcripts in the new assembly. Figure S2 A comparison of assembled transcripts read count. The boxplots represent the range between the 1st and 3rd quartiles of the data by the coloured boxes, the median is the horizontal bar and points shown beyond the whiskers are >95% of the data. Table S3 Transrate good contigs. Table S4 Olignucleotide sequences. Table S5 Alignment with DNA sequence of PCR product. (DOCX 787 kb) [file 12864_2017_3505_MOESM1_ESM.docx]

**Removal of bacterial contigs**

Using the GC content, read depth and best phylogenetic match information for each transcript it was possible to determine criteria to remove the most likely contaminant sequences. Figure S1 shows the output from TAGC for both *D. lacteum* and *D. fasciculatum* prior and after removal of the unwanted transcripts. By examining Figure S1 we determined that all transcripts with GC content ≥ 50% and read count ≤ 10 were likely bacterial contaminants. This is justifiable as Dictyostelid species typically have a low GC content (around 30%, see [[1](#_ENREF_1)]) and it is expected they would be more highly expressed than bacterial transcripts. After removing the transcripts which belonged to the different bacterial taxonomic groups, the remaining unannotated transcripts were filtered on the basis of the GC and read count criteria. In total 59,100 *D. fasciculatum* and 57,145 *D. lacteum* Trinity transcripts were reduced to 46,779 and 38,508 respectively.


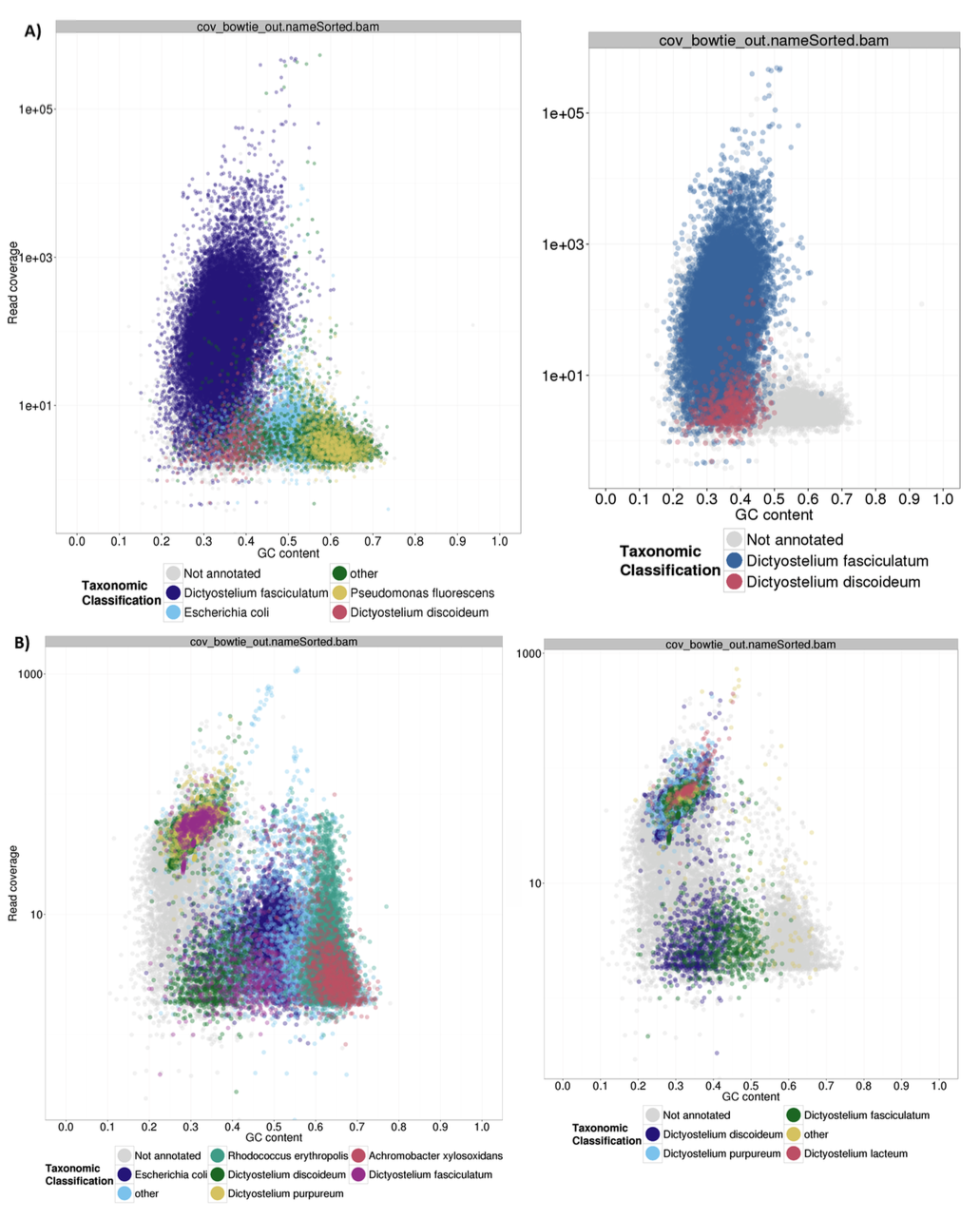


Figure S1. **TAGC plot for *D. fasciculatum*** (A) and *D. lacteum* (B) before and after filtering. Each colour blob represents different taxa with unmatched transcripts are shaded in grey. The unannotated grey coloured transcripts after filtration set further filtered by high GC and low read coverage. This plot shows a major blob of transcripts that are annotated with the Dictyostelium fasciculatum species with high coverage and lower GC content. Other contaminations form E.coli, pseudomonas fluorescence and other species has also been highlighted with different colours. These contaminations clearly make different blobs with lower read coverage and high GC content. However, it’s good to see that there are some other transcripts that showing matched to dictyostelium discoideum- that clearly reflect the presence of some novel unannotated transcripts in the new assembly.

**Transcript Read Depth**

Figure S2 shows the read depth distributions for all transcripts within each species. Here we see that the four species have similar median read evidence for each transcripts, *D. discoideum* has the lowest median read depth per transcript (233) and *P. pallidum* the highest (552). The distribution for *D. fasciculatum* appears to be different from the other three possibly due to its raw data not being normalised whereas the others were.


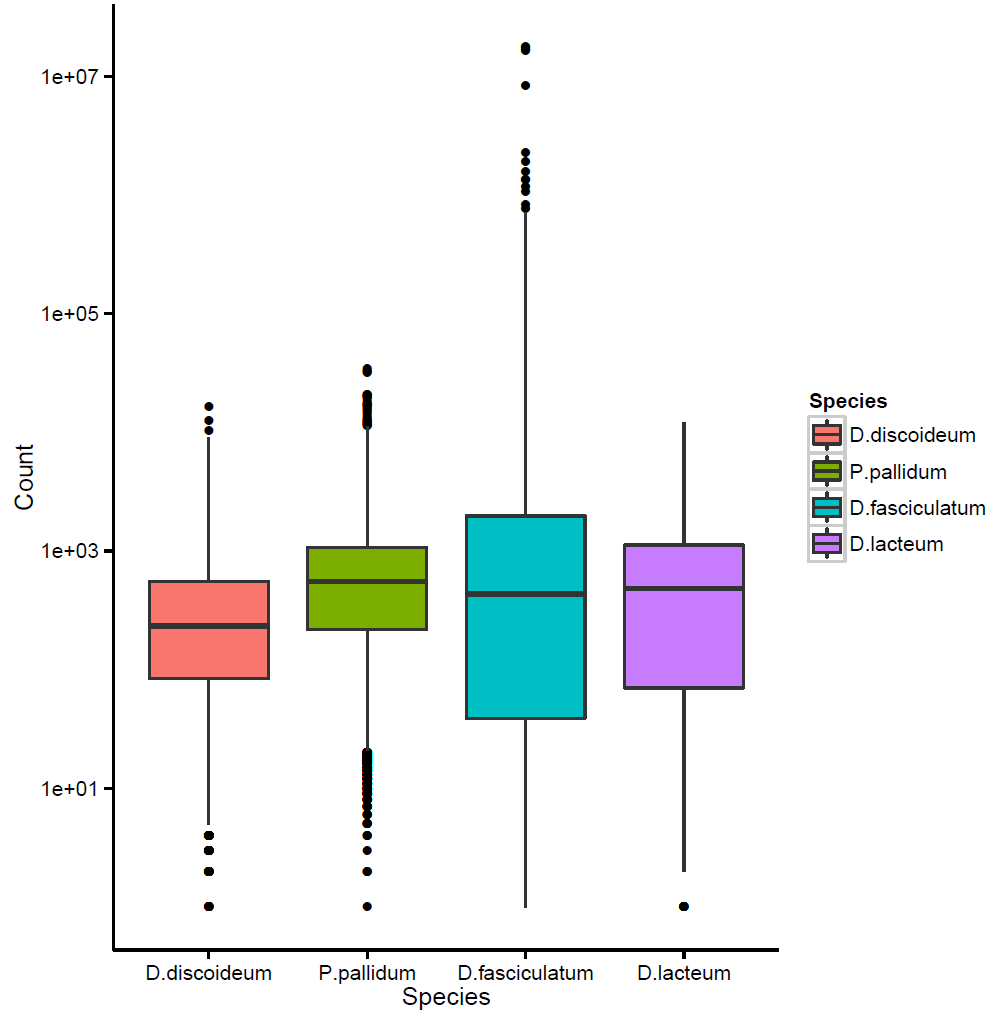


Figure S2. **A comparison of assembled transcripts read count.** The boxplots represent the range between the 1^st^ and 3^rd^ quartiles of the data by the coloured boxes, the median is the horizontal bar and points shown beyond the whiskers are >95% of the data.

**Table S3. Transrate good contigs**

| **Transcript Set** | **Bad Contigs** | **Good Contigs** | **Good Contigs (%)** |
| --- | --- | --- | --- |
| *D. discoideum* PASAua | 2,321 | 9,202 | 79.9 |
| *D. discoideum*  CDS | 3,317 | 8,966 | 73.0 |
| *P. pallidum*  PASAua | 1,930 | 10,919 | 85.0 |
| *P. pallidum*  CDS | 2,405 | 9,035 | 79.0 |
| *D. lacteum*  PASAua | 999 | 10,316 | 91.2 |
| *D. lacteum*  CDS | 986 | 9,246 | 90.4 |
| *D. fasciculatum* PASAua | 353 | 12,361 | 97.2 |
| *D. fasciculatum*  CDS | 1,347 | 10,532 | 88.7 |

**Table S4. Olignucleotide sequences**

| **Trancript** | **DNA sequence** |
| --- | --- |
| comp5787_c28_seq1F | TACAAGGTTTAGGATCcCCAAAATATG |
| comp5787_c28_seq1R | TACAGGTTGCTCGAGCTGGTGTAATAC |
| comp5569_c2_seq1F | TTGATAAAGCATATGGaTccTTAGATTGA |
| comp5569_c2_seq1R | TGTGATcTCGAGAAGTTAAAATATTTAGT |
| comp1545_c0_seq1F | TGGTTGTAAAGGgATcCCATTTTGTTC |
| comp1545_c0_seq1R | TGTCATAAATTcTcGAGATTATTTGGTAT |
| comp5953_c11_seq1F | AGTTGTTGGTGCAACTTGTGgaTCcTCAC |
| comp5953_c11_seq1R | ACCATTATCTcGagTTGATGATATTGAAG |
| comp5953_c48_seq1F | ATGATATTTGGATccTATCAACATCAACA |
| comp5953_c48_seq1R | ACACTACCAACtCGAgTAAATTCATT |
| comp2066_c1_seq1F | ACAACAACAACAAggATCcTCATTAG |
| comp2066_c1_seq1R | ATGAACTTTTAAATTCtCgAG |
| comp6065_c2_seq1F | TAGgGAtcCAATTTACGATACATGTA |
| comp6065_c2_seq1R | TAACTGTTGCTcGagCTGAAAAAGG |
| comp470_c0_seq1F | TGGGGAATGGaTcCTATCGAGTATAC |
| comp470_c0_seq1R | ACTATCAcTCgAgACTGTACAAAGTG |
| comp4678_c1_seq1F | ATGGGTGAGTgGaTCCTAAGCCTTGGGTG |
| comp4678_c1_seq1R | TACGGACAATAATCtcGAGCCAGCATAGC |
| comp4660_c0_seq1F | ATGAAAGAAggATCcATAGAAGAAG |
| comp4660_c0_seq1R | TCTAATAGTTTCATCTcTCGAgGGATC |
| comp4660_c4_seq1F | ACATTTATTGGATccAAATTATCCATAC |
| comp4660_c4_seq1R | TGTTTTCTcgAgCCATGAAAAATTGTG |

Forward (F) and reverse (R) oligonucleotide primers harbouring BamHI and XhoI restriction sites (underlined), respectively. The nucleotides in lower case were altered to generate the restriction sites.

**Table S5. Alignment with DNA sequence of PCR product**

|  | Alignment with PCR product | | |
| --- | --- | --- | --- |
| Transcript | mismatch (nt) | introns | product length (nt) |
| comp5787_c28_seq1 | 4 | 2 | 924 |
| comp5569_c2_seq1 | 7 | 0 | 1491 |
| comp1545_c0_seq1 | 0 | 0 | 758 |
| comp5953_c11_seq1 | 2 | 0 | 646 |
| comp5953_c48_seq1 | 4 | 0 | 657 |
| comp6065_c2_seq1 | 0 | 0 | 477 |
| comp4660_c0_seq1 | 0 | 0 | 574 |
| comp4660_c4_seq1 | 1 | 0 | 597 |

The products, amplified from genomic DNA, were sequenced in both directions. Sequences were aligned with the transcript sequences and mismatched nucleotides and introns were counted.

1. Heidel AJ, Lawal HM, Felder M, Schilde C, Helps NR, Tunggal B, Rivero F, John U, Schleicher M, Eichinger L *et al*: **Phylogeny-wide analysis of social amoeba genomes highlights ancient origins for complex intercellular communication**. *Genome research* 2011, **21**(11):1882-1891.
