# Supplementary figures and images for: Improved annotation with de novo transcriptome assembly in four social amoeba species
Source: BMC Genomics. 2017 Jan 31;18:120. doi: 10.1186/s12864-017-3505-0 (PMC5282741; doi:10.1186/s12864-017-3505-0)

DDB\_G0267384

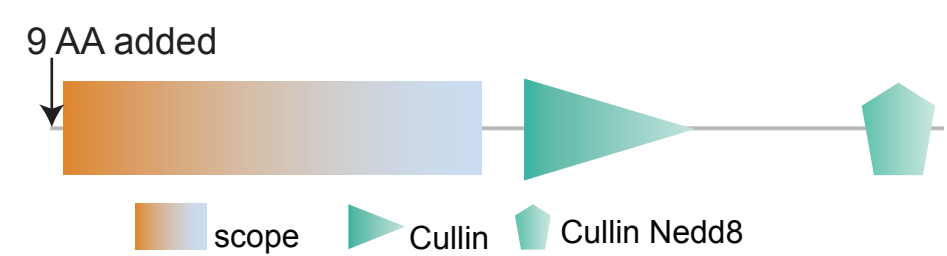

DDB\_G0269244

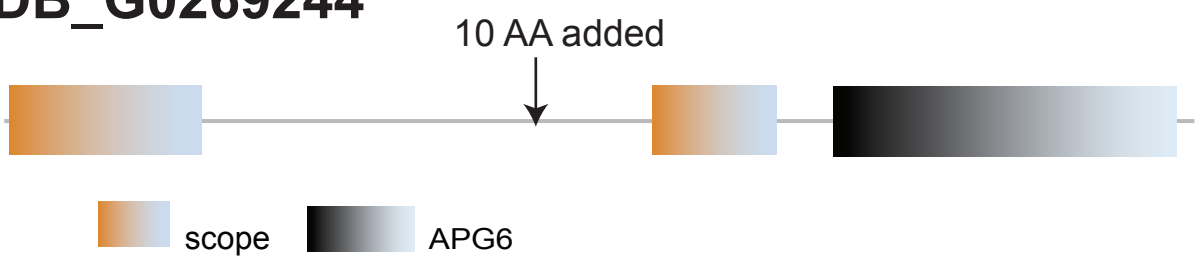

DDB\_G0274387

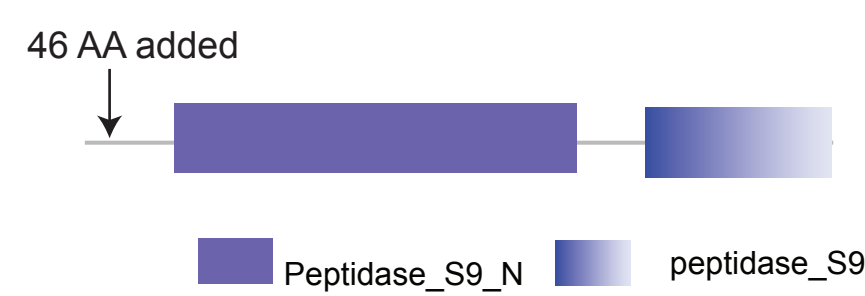

DDB\_G0279409

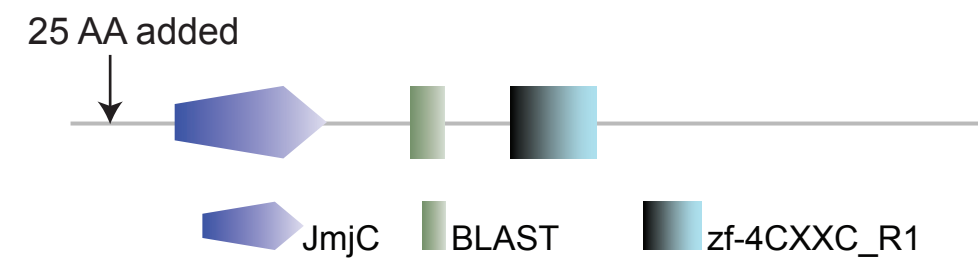

DDB\_G0282291

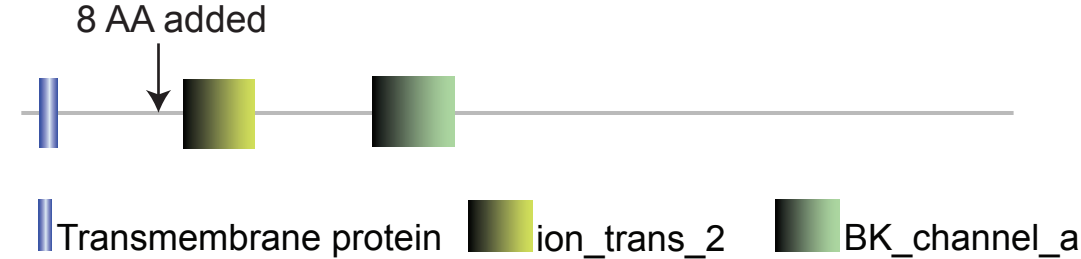

DDB\_G0289121

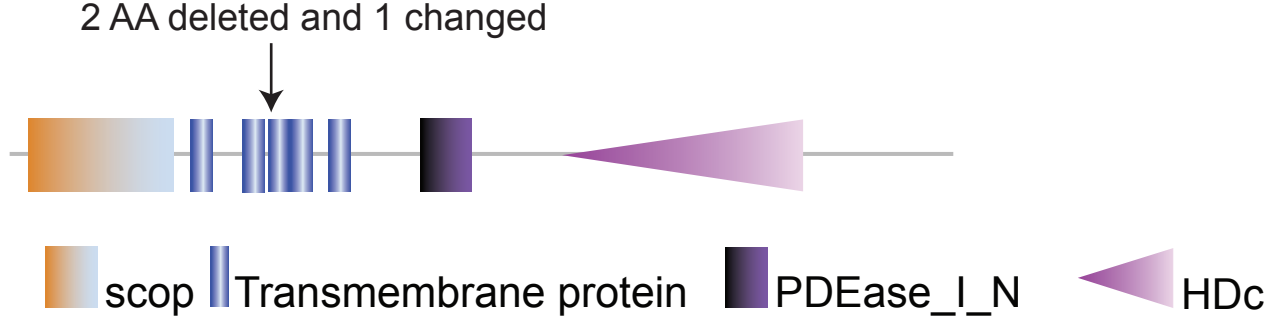

DDB\_G0291239

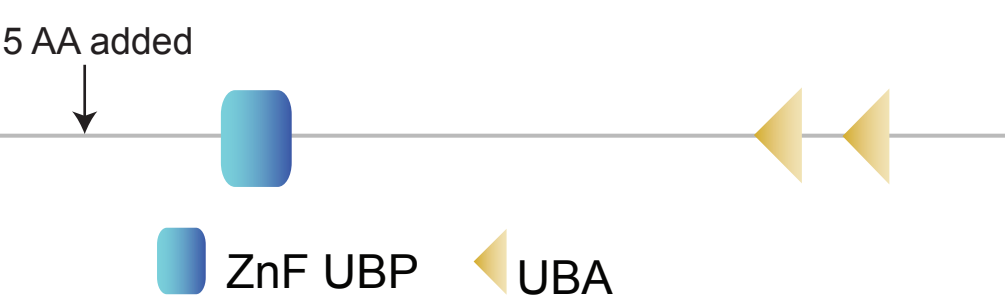

DDB\_G0268920

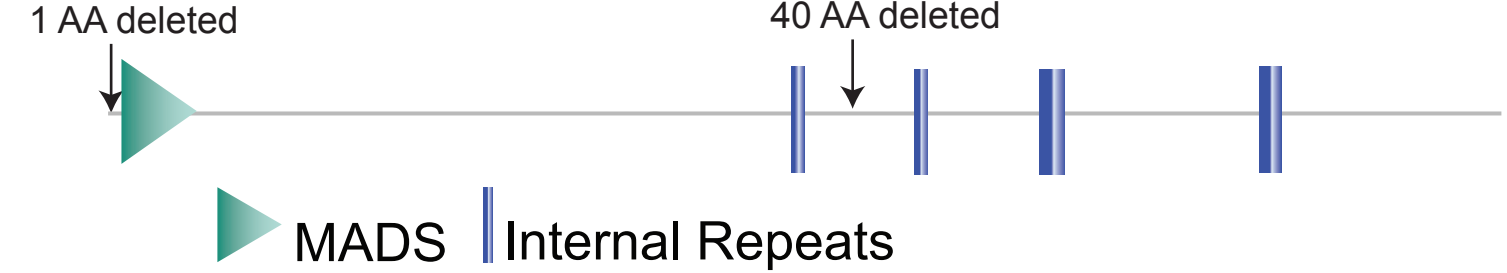

DDB\_G0271502

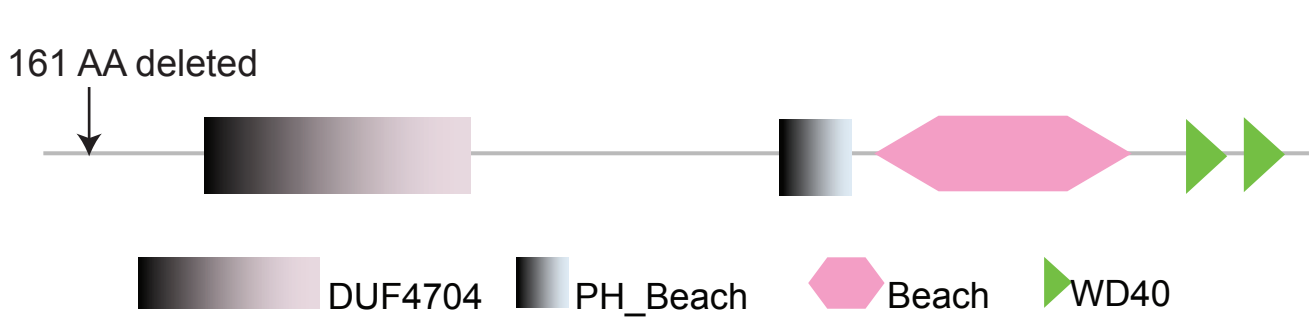

DDB\_G0274577

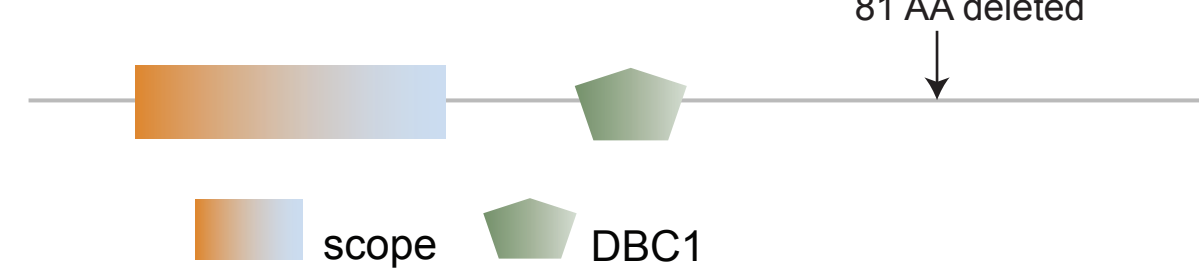

DDB\_G0275445

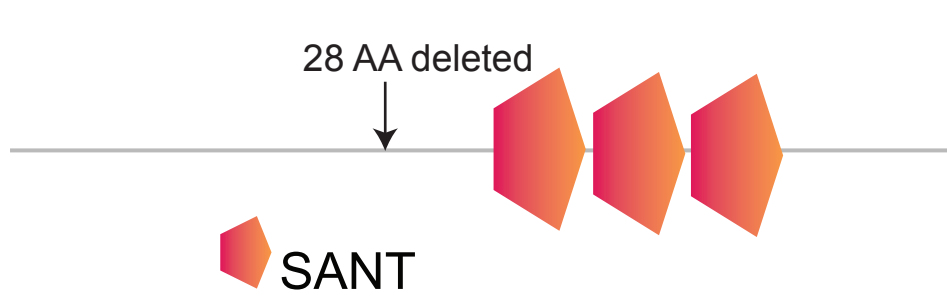

DDB\_G0269160

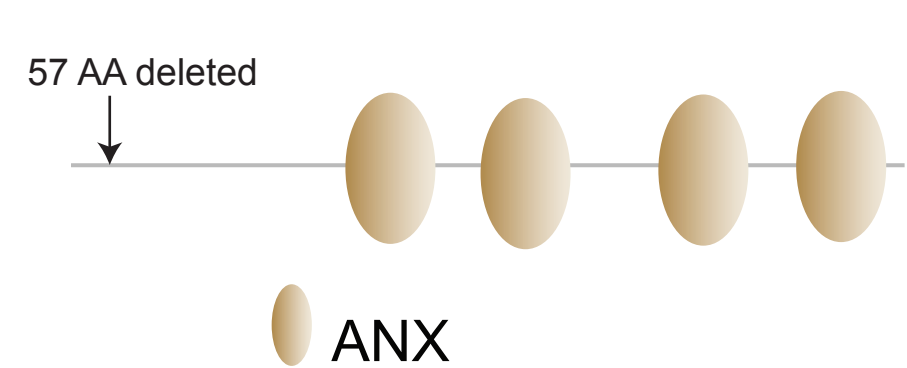

DDB\_G0274607

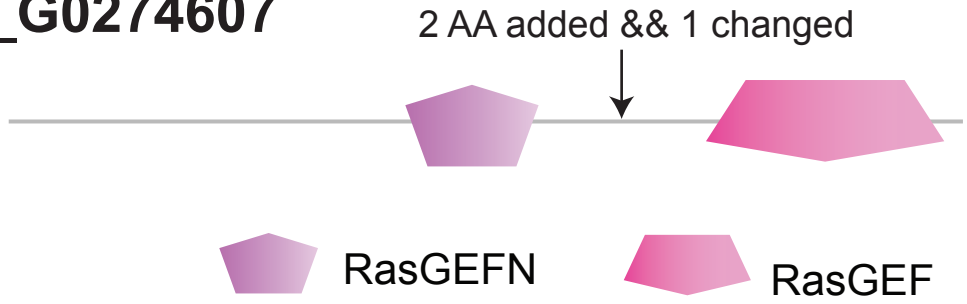

DDB\_G0290453

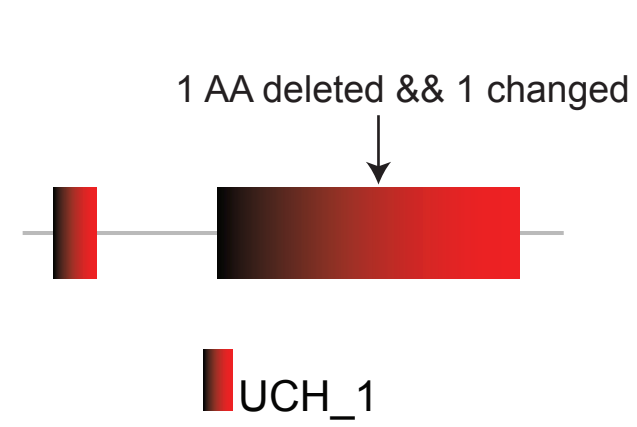

DDB\_G0277719

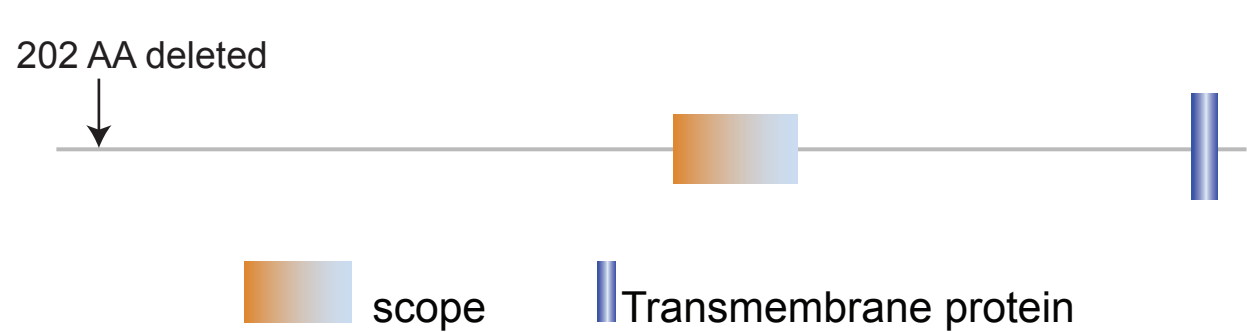

Supplement: Additional file 2: Figure S3. — Annotated domain diagrams for 16 D. discoideum developmentally relevant proteins which have had their protein sequence altered. (PDF 367 kb) [file 12864_2017_3505_MOESM2_ESM.pdf]
